# Theoretical Investigations into the Different Properties of Al-Based Fluoroperovskite AlMF_3_ (M = Cr, B) Compounds by the TB-MBJ Potential Method

**DOI:** 10.3390/ma15175942

**Published:** 2022-08-28

**Authors:** Hukam Khan, Mohammad Sohail, Rajwali Khan, Nasir Raman, Asad Ullah, Aurangzeb Khan, Abed Alataway, Ahmed Z. Dewidar, Hosam O. Elansary, Kowiyou Yessoufou

**Affiliations:** 1 Department of Physics, University of Lakki Marwat, Lakki Marwat 28420, Khyber Pakhtunkhwa, Pakistan; 2 Department of Mathematics, University of Lakki Marwat, Lakki Marwat 28420, Khyber Pakhtunkhwa, Pakistan; 3 Department of Physics, Abdul Wali khan University Mardan, Mardan 23200, Khyber Pakhtunkhwa, Pakistan; 4 Prince Sultan Bin Abdulaziz International Prize for Water Chair, Prince Sultan Institute for Environmental, Water and Desert Research, King Saud University, Riyadh 11451, Saudi Arabia; 5 Department of Agricultural Engineering, College of Food and Agriculture Sciences, King Saud University, Riyadh 11451, Saudi Arabia; 6 Plant Production Department, College of Food & Agriculture Sciences, King Saud University, Riyadh 11451, Saudi Arabia; 7 Department of Geography, Environmental Management, and Energy Studies, University of Johannesburg, APK Campus, Johannesburg 2006, South Africa

**Keywords:** Density Functional Theory, fluoroperovskite, optical properties, structural properties, electronic properties

## Abstract

Al-based fluoroperovskites compounds AlMF_3_ (M = Cr, B) are investigated computationally and calculated their elastic, structural, optical, and electrical properties in this study utilising TB-MBJ potential (also GGA+U for AlCrF_3_) approximations, according to the Birch Murnaghan Equation curve and tolerance factor, these material are structurally cubic and stable. The IRelast algorithm is used to forecast elastic properties, and the outputs show that these compound are mechanically stable, anisotropic and ductile. AlBF_3_ has a metallic nature and overlapping states, while AlCrF_3_ have a narrow indirect band gap at (X-M) points of symmetry, with band gaps of 0.71 eV for AlCrF_3_ and zero eV for AlBF_3_. The partial and total density of states are being used to determine the influences of different basic states to the conduction and valence bands (TDOS & PDOS). Investigation of Optical properties shows that these compounds have low refractive index and high absorption coefficient, conductivity, reflective coefficient at high energy ranges. Owing to the indirect band gap, the applications of these compounds are deemed in conducting industries. Here we are using these compounds for first time and are examined using the computational method, which delivers a complete view into the different properties.

## 1. Introduction

Fluoroperovskites compounds are familiar and having the natural chemical formula ABF_3_, where cations are “A” and “B” with positive and Fluorine element having negative charge is an anion. Fluoroperovskites are a fascinating group of materials with a stable mechanically crystal structure and good optoelectronic characteristics ranging from semiconducting material having energy ranging from 1–4 eV and insulating materials with energy (above 4 eV) in nature. Fluoroperovskites have received a lot of interest recently due to its usage in a variety of industries, including semiconductor production, scintillation materials, radiation dosimeters and lenses in optical lithography [1,2,3]. Experimentally and computationally by a various authors have been intensively researched on Fluoroperovskites compounds [4,5,6,7] due to their vast range of application.

Fluoroperovskites frequently have a large band gap of energy [8,9,10,11]. The large energy band gap in these compounds marks them technologically significant. When Fluoroperovskites doped with lanthanide Er and Ce ions, such as is the case with BaLiF_3_ and KMgF_3_ show promise as a scintillation and radiation dosimeter [9]. BaLiF_3_ and KMgF_3_ are employed in optical lithography enhancers as UV vacuum materials for optics [7,11,12,13,14,15,16,17,18,19,20,21,22,23,24]. Murtaza et al. [5] conducted A hypothetical study conducted on a compound based on Ag fluoroperovskites AgMgF_3_ and AgZnF_3_. The proposed compounds are suitable for a wide range of requests in current devices due to their projected large absorption energy spectrum. The optoelectronic properties of the said Sn-based fluoro-perovskites were inspected, and these composites were exposed to be insulators, with the expected Auger-Free luminescence (AFL) [25,26]. TlMnX_3_ (X) exhibits optoelectronic, structural, and magnetic characteristics [27,28]. Several investigations have indicated that for use in radiation detection, thulium-based compounds are being developed [29,30]. Because in these composites, a thallium atom exists—due their effectively large atomic number, it improving detection efficiency. Additionally, because they only require one growth condition, the compounds’ straightforward cubic shape makes them a technologically promising contender. The TB-MBJ methodology is used to check the structural, elastic, optical, and electrical properties of AlMF_3_ (M = Cr, B) compounds in depth. We observed that AlCrF_3_ over a short range of energies is an excellent electrically conductor, making it a practical option for conduction in electrical uses, using the computer simulation tool wien2k. AlCrF_3_ has been discovered to be an electrical conductor with great transparency over a narrow energy range, making it an excellent choice for electrical applications. Despite the interest in fluoroperovskites for a variety of applications, there is no literature on the research of Al-based fluoroperovskites to our knowledge.

This research has four primary components. In Section 1, the compounds chemical structure are explained, the calculating method is explained in Section 2, Results and discussions are covered in Section 3 while the conclusions are covered in Section 4.

## 2. Materials and Methods

**Figure 1:**
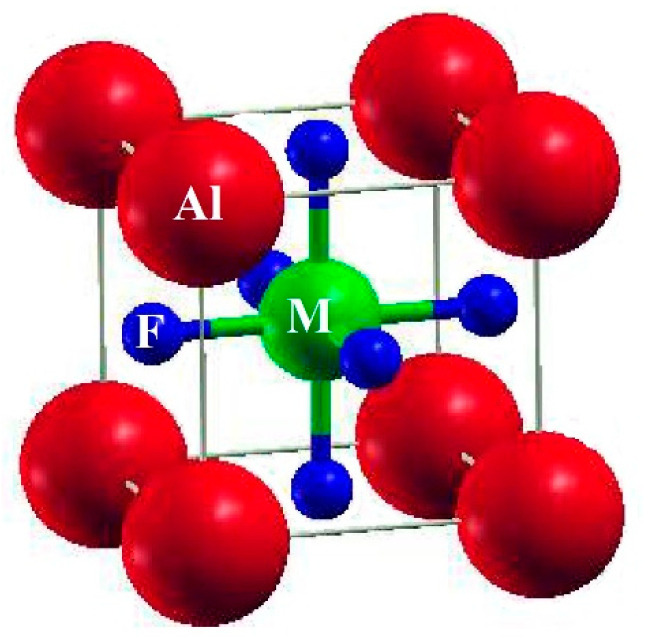
Figure represents the AlMF_3_ (M = Cr, B) fluoroperovskite unit cell structure.

The ternary fluoroperovskite AlMF_3_ (M = Cr, B) compounds are cubic-structure perovskites with the space group Pm-3m (#221). The unit cell contains one molecule with Al and M (M = Cr, B) at the (0, 0, 0) and (1/2, 1/2, 1/2) Wyckoff coordinates, respectively, and three Fluorine atoms (Figure 1) located at (1/2, 0, 1/2), (0, 1/2, 1/2), and (1/2, 1/2, 0). Density Functional Theory has a common framework that is utilized in simulation studies to examine elastic, electrical, and optical properties. WIEN2K code can be utilized to maximize the utility of the FP-LAPW technique [31]. The calculation was carried out using the mBJ for AlMF_3_ (M = Cr, B). Simultaneously, the GGA + U method was applied for the AlCrF_3_ due to it, containing a transition element. The effective Hubbard parameter (Ueff) in the AlCrF_3_ system was adjusted to 4 eV for the Cr 3d orbital, which yields acceptable results for the band gap, magnetic moments, and optical properties [32,33].

**Figure 2:**
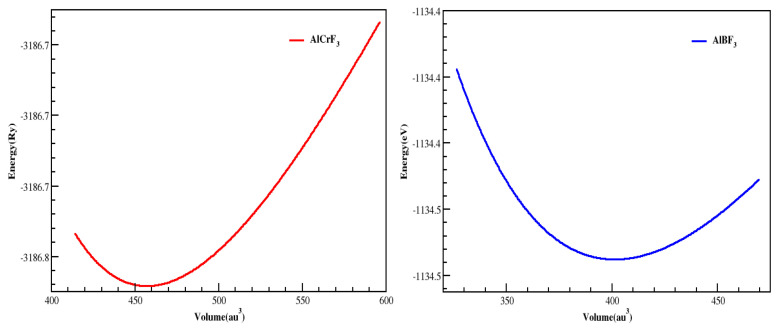
AlMF_3_ (M = Cr, B)-based cubic perovskites and their total energy dependency are represented.

The energy-versus-volume graph in Figure 2 is based on the Murnaghan equation of state, which determines the structural features of compounds [34]. An appropriate value of RMT was chosen for this inquiry to ensure no charge leakage from the total and core energy.

The RMT values for Cr, B, and Al were 1.74, 1.74, and 2.5, respectively, while the values of RMT for F in the AlCrF_3_ and AlBF_3_ were 1.77 and 1.74, respectively. In the Muffin tin spheres, the wave function is stretched in harmonics up to l_max_= 10 because there are 2000 K-points and 12 G_max_. The valence bands and core had a quite large 6.0 Ry energy difference.

## 3. Results

### 3.1. Electronic Properties

**Figure 3:**
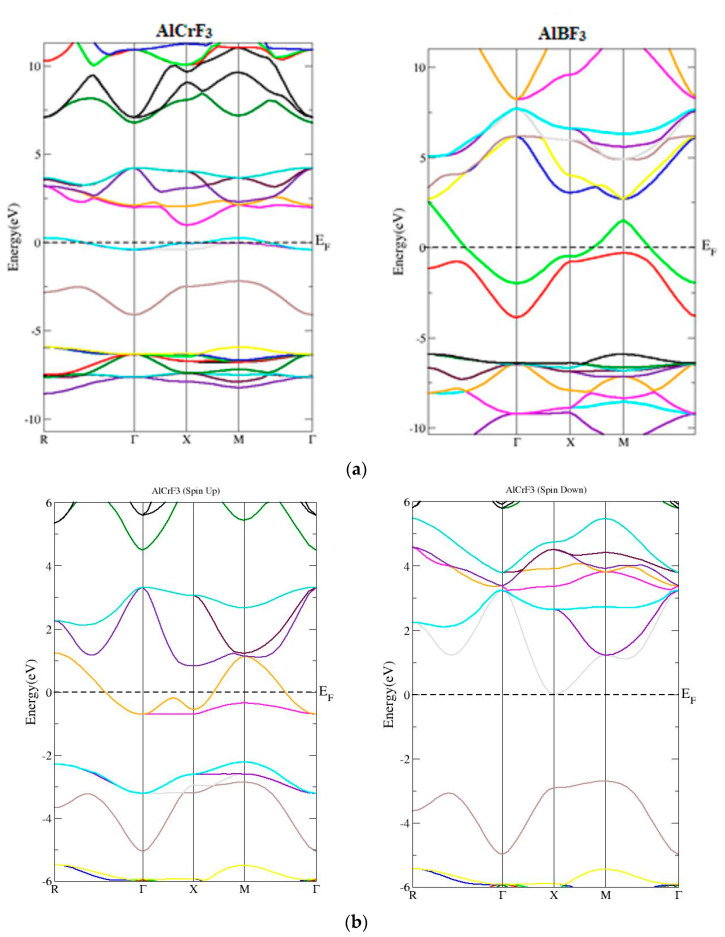
(**a**) Computed band structures of AlMF_3_ (M= Cr, B) using TB-MBJ. (**b**) Computed band structures of AlCrF_3_ using GGA + U.

In this unit, the energy bands structures and DOS of compounds under investigation are reported. In the primary Brillion area of AlMF_3_ (M=Cr, B), the estimated bands structure and symmetry directions are shown in Figure 3. Since no zero energy should exist at or close to the maxima location of the valence band. Using the said potential approach, the valence band maxima of the compound AlBF_3_ overlap the minima of conduction band and zero band gap while the band gaps for the compounds AlCrF_3_ were determined to be 0.71 eV, respectively. The valence band maximum in both cases crosses the fermi energy level, indicating that both compounds have metallic properties. The indirect band nature of AlCrF_3_ is shown by the electronic band structure in Figure 3a, which shows that the conduction group of states minima of AlCrF_3_ occur at the equilibrium point X and the valence band peak occur at the point M (a).The metallic character of the AlCrF_3_ combination is obvious. Simultaneously the GGA + U were applied for the AlCrF_3_, due to the transition element. The effective Hubbard parameter (U_eff_) in the AlCrF_3_ system was adjusted to 4 eV for the Cr-3d orbital. That shows that the nature of the material changed from metallic to half metallic nature (see Figure 3b). The minima of conduction band of AlBF_3_ and the maxima of valence band occur at R of Brillion Zone, which demonstrates the direct band gap. The energy band structures and DOS of the compounds of interest are described in this section. Figure 3 shows the computed AlMF_3_ (M=Cr, B) band structure, as well as the symmetries orientations within the equilibrium geometry within the first Brillion region.

### 3.2. The DOS (Density of States)

**Figure 4:**
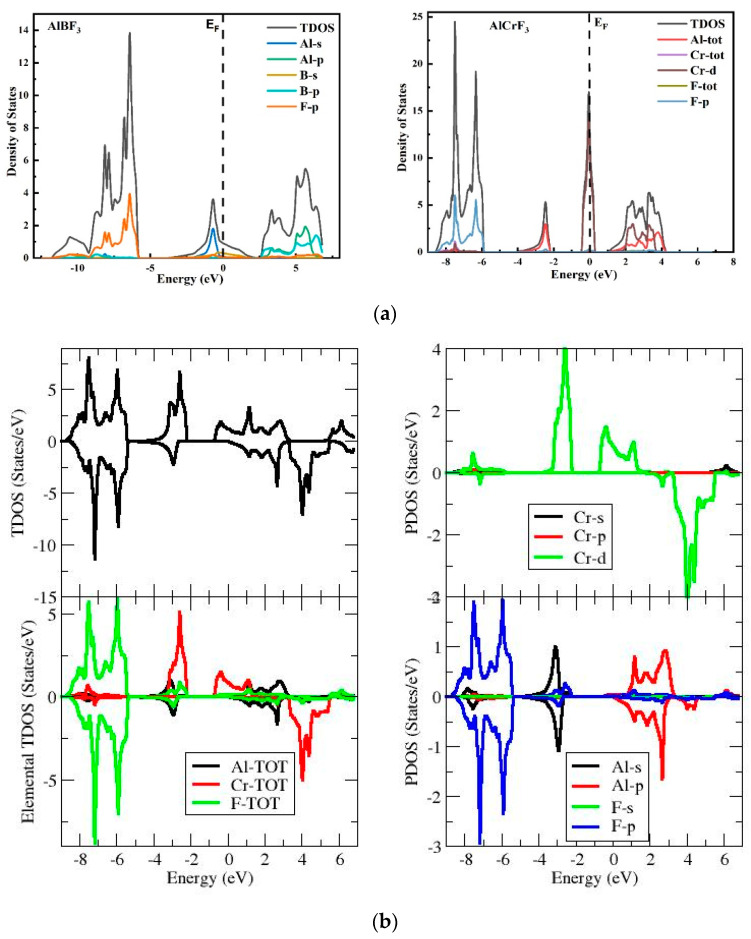
(**a**) Represents TDOS and PDOS of ternary AlMF_3_ fluoroperovskites (M = Cr, B) using GGA. (**b**) Represents TDOS and PDOS of ternary AlCrF_3_ fluoroperovskites using GGA + U.

Figure 4a depicts the electronic states contributions using the T-DOS and P-DOS (total density and partly density of states) to the VB and CB bands for both compounds under inspection. As depicted as EF, the vertical dotted line represents Fermi level. The left-hand side of Fermi levels is valance band while on the right of the Fermi level is conduction band. The various states of the constituent materials are used to gain a better understanding. The plots of total DOS and partial DOS for AlCrF_3_ show that the VB is governed by Al-tot, Cr-tot and F-tot due to the superposition of all states, but F-s, Cr-s, Cr-p, Al-s, Al-p states do not contribute. The Al and Cr element plays a crucial role in CB while in the valence band the most impact comes from the elements F and Al. In case of AlBF_3_, the VB is led by F-p states, with Al-s and Al-p states contributing only minimally. In conductional band for the compound AlBF_3_ most contribution come from the states Al-p and B-p while F-p and B-s take part only in small amount.

TDOS and PDOS for the stress-free AlCrF_3_ compound with and without incorporating Hubbard correction U were performed in order to examine the influence of the Hubbard U constraint on the stress-free electronic structure (Figure 4b). The spin of majority states are pushed up close to the FL when Hubbard correction is used, but the spin of minority states become less localized close to and above the FL. As a result, utilizing GGA+U, the AlCrF3 structure becomes half metallic.

Although Cr atoms exhibit net up and down magnetic moments, as shown in Figure 2b,c, it is important to keep in mind that, close to the Fermi level, the Cr atoms mostly contribute in the valance band in up spin states and in the conduction band in down spin states. The predicted magnetic moment for the Cr atom is around 3.40620 B.

### 3.3. Elastic Properties

Constants of Elasticity Cij, which are important and necessary, can be practiced to characterize elastic properties of materials. The significant properties of the compounds are elastic characteristics which help in finding the stress reaction, applying the macroscopic stress to the chemicals compounds. The elastic constants describe how a material deforms under tension and then returns to its initial shape once the stress has removed [35]. These elastic constants can be used to explain structural stability, atomic bonding properties, and isotropic or anisotropic properties in the atomic sphere. The three different and self-regulating elastic coefficients for a cubic system are C_11_, C_12_, and C_44_. C_ij_ is determined by tilting the unit cell with the proper strain tensor to create an energy strain relation (Table 1). IRelast-package [36], which Jamal Murtaza advanced, is used and executed inside the WIEN2k code in this work. The ideal cubic crystal structure of every Al based fluoroperovskite in this study as revealed in Figure 1. The relationship between energy and volume deviation is depicted in Figure 2. The Fitting the Birch-Murnaghan equation of states provides the lattice coefficients at equilibrium [37,38,39,40,41].

The reaction caused by the application of force to a crystal using its elastic factors was calculated, which gives a lot of information about the mechanical properties of the material. C_11_, C_12_, and C_44_ were used to compute the elastic properties of the cubic symmetric crystals, including their stiffness and stability (three self-governing elastic factors). The computed elastic constant (C_ij_) values are shown in Table 2.

The experimental values were compared to the elastic constants discovered for AlCrF_3_. The elastic constants for AlCrF_3_ (79.11 GPa, 44.85 GPa, 11.86 GPa) and AlBF_3_ (77.00 GPa, 94.23 GPa, 31.92 GPa) were computed using DFT.

The study of AlCrF_3_ showed that the TB-MBJ approximation potential method provides comprehensive, extra-exact elastic characteristics that are nearer to the experimental results than using the GGA-WC method. Using the calculation, we can obtain the (*B*) bulk modulus from constants of elasticity using their relationships (ECs). The ECs were all positive except for C_44_ and met the criteria. *C_11_* > 0; *C_44_* > 0; *(C_11_* + *2C_12_*) > *0*; *(C_11_-*C_12_) > 0; *C_12_ >B > C_11_* for flexible stability [37].

Table 2 displays the outcomes of the following relationships when applying Young’s modulus (*E*); the Anisotropy factor (*A*), Poisson’s ratio (*v*), and Pugh’s index ratio (*B/G*) are obtained.

(1) $$B=\frac{C_{11}+2C_{12}}{3}$$

(2) $$A=\frac{2C_{44}}{C_{11}-C_{12}}$$

(3) $$E=\frac{9\mathrm{BG}}{G+3B}$$

(4) $$v=\frac{3B-2G}{2\left(G+2B\right)}$$

(5) $$G_{v}=\frac{C_{11}-C_{12}+3C_{44}}{5}$$

(6) $$G_{R}=\frac{5C_{44}\left(C_{11}-C_{12}\right)}{4C_{44}+3C_{11}-C_{12}}$$

(7) $$G=\frac{G_{v}+G_{R}}{2}$$

Fracture resistance is offered by the bulk modulus B, while plastic deformation occurs due to the shear modulus G resistance. The *B/G* ratio determines the brittleness or ductility of a substance [24]. If the value of *B/G* is larger than 1.75, it exhibits ductility, and it exhibits brittleness if the *B/G* is less than 1.75; so, both materials showed ductility because the ratio of B and G (Pugh’s criterion) value was greater than 1.75. Poisson’s v ratio is a measure of a material’s brittleness [25]. However, the materials were both ductile, as illustrated in Table 2, and would be ductile if *v* was more than 0.26; otherwise, they would be brittle. The elastic anisotropy factor “A.” must be equivalent to one; otherwise, the substances would be isotropic. The deviation from one tells us how elastically anisotropic the crystal is, to a certain extent. We report that the value of A for both of our compounds was different from one, indicating that they are all anisotropic. The best indicator of a material’s stiffness is Young’s modulus (E). For a given substance, when Young’s modulus is high, the material is stiffer, and both compounds showed the stiffest property. More than any other elastic attribute, the bonding power as measured by Poisson’s ratio reveals the bonding power’s uniqueness.

### 3.4. Optical Properties

The optical characteristics of the solid materials were designed using the TB-MBJ approximation potential technique. Characterizing electronics’ complex dielectric function for solid materials, i.e., *ε*(*ω*), allows us to learn about their optical properties. Both inter-band and intra-band transitions are important in conveying this complex function. Contributions from inter-bands are particularly important for metals [38] and are classified as direct or indirect transitions. Due to their low contribution to *ε*(*ω*) and their phonon scattering nature, we neglected indirect inter-band transitions. An electronic system’s linear response to the application of an exterior electric field is provided by the anisotropic property of the material with the dielectric function *ε*(*ω*), which is just a complex form of the second-order symmetric tensor. Ehrenreich and Cohen’s equation define a complex dielectric function that can help to find the ocular properties of a compound [34].

(8) $$\varepsilon \left(\omega \right)=\varepsilon_{1}\left(\omega \right)+i\varepsilon_{2}\left(\omega \right)$$

The *ε*_1_(*ω*) and *ε*_2_(*ω*) are real and non-real components, respectively. The real portion of the dielectric permittivity can be resolved from the imaginary portion, and it only depends on *ω*′^2^–*ω*^2^, which provides the integral denominator; it also has a direct correlation with the energy gap Eg. By the application of the Kramers–Kroning relationship, the imaginary part can be extracted from the real part as:

(9) $$\varepsilon_{2}\left(\omega \right)=\frac{8}{2\pi \omega }\sum_{nn^{\prime }}\int {\left|P_{nn^{\prime }}\left(k\right)\right|}^{2}\frac{dS_{k}}{\nabla \omega_{nn^{\prime }}\left(k\right)}$$

The real portion *ε*_1_(*ω*) can be obtained by using the Kramers–Kronig relationship, given as:

(10) $$\varepsilon_{1}\left(\omega \right)=1+\frac{2}{\pi }P\int_{0}^{\infty }\frac{\omega^{\prime }\varepsilon_{2}\left(\omega \prime \right)}{\left({\omega^{\prime }}^{2}-\omega^{2}\right)}d\left(\omega^{\prime }\right)$$

For the material response to incident light, the incident optical energy series is 0–14 eV is used. The dispersive effects are demonstrated using a genuine part of the material’s surface, whereas light absorption is demonstrated using a model.

#### 3.4.1. Refractive Index

**Figure 5:**
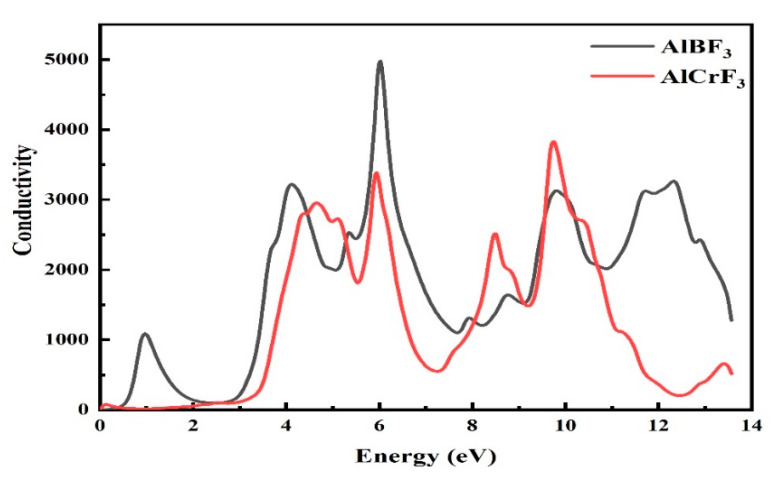
Figure represents the conductivity of the given AlMF_3_ compounds (M = Cr, B).

The fictitious region exhibits optical transitions and light absorption within the energy bands. Other optical properties including the absorption coefficient, refractive index, optical conductivity, extinction coefficient, and reflectivity can be calculated using the dielectric functions [28]. The conductivity of the compound AlCrF_3_ increased with the energy range 0 to 14 eV, as seen in Figure 5. AlCrF_3_ and AlBF_3_ had maximal optical conductivity values of 9.8 eV and 6.0 eV, respectively.

**Figure 6:**
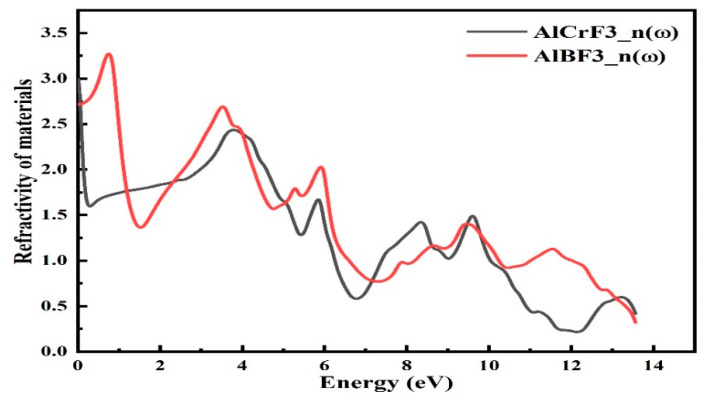
Figure represents the Refractive Index n(w) for the AlMF_3_ (M= Cr, B) compounds.

The refractive index of a material tells us that how fast light flows through the material. Some formulae [29] can be used to calculate the refractive index. We can see in Figure 6 that the refractive index “n” of the compound AlCrF_3_ had a maximum value of 3.25 at 0.4 Ev and the compound AlBF_3_ had a maximum value of 3.0 at 0 eV, respectively. The refractive index value for the compound AlCrF_3_ was 2.75 at 0 eV energy, gradually declining with various peaks. The maximum refraction of the incident light occurred at 0 eV for the compound AlBF_3_ and then gradually decreased. Different peaks at 3.25, 2.55, 2.0, and 1.5 eV were seen for AlCrF_3_, whereas AlBF_3_ had peaks at 3.0, 2.44, 1.6, and 0.5 eV, as shown in Figure 6.

As the incident photon energy increased, in both cases, the value of the refractive index decreased at 14 eV; most of the light passed through the material and it acted as transparent material, as shown in Figure 6.

#### 3.4.2. Absorption Coefficient

The absorption constant of each substance tells us how these substances would react to radiation. The frequency affects how photons interact with electrons and cause electrons to travel from the valence region to the conduction region grounded on the absorption constant. The absorption coefficient is a measurement of a material’s capacity to absorb a specific energy photon [30]. The absorption coefficients support both the real component as well as the imaginary components of the dielectric functions. This is how it is written:

(11) $$I\left(\omega \right)=\sqrt{2}\omega {\left[\sqrt{\varepsilon_{1}^{2}\left(\omega \right)+\varepsilon_{2}^{2}\left(\omega \right)}-\varepsilon_{1}^{}\left(\omega \right)\right]}^{1/2}$$

**Figure 7:**
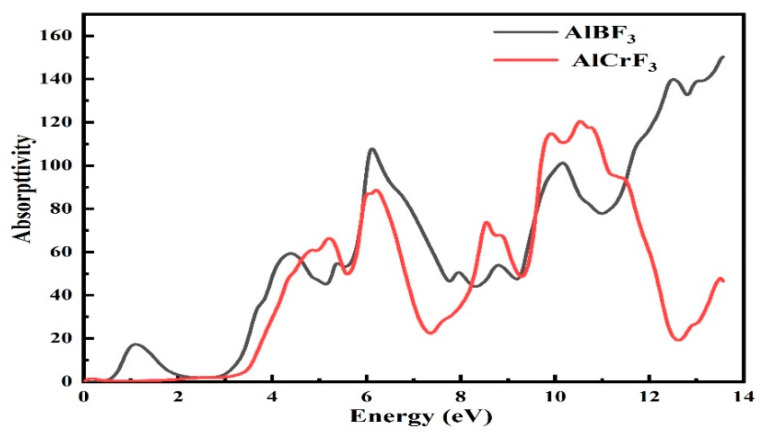
The Computed Absorptivity (absorption coefficient) for the fluoroperovskite AlMF_3_ (M = Cr, B).

The computed absorption coefficients of AlCrF_3_ and AlBF_3_ are shown in Figure 7. The absorption section of the AlCrF_3_ spectra began at 2.0 eV and the peak value was 120 at 10.5 eV, while the absorption coefficient for the compound AlBF_3_ spectra began at 0.5 eV and peaked at 140.0 at 13.6 eV, as shown in Figure 7.

#### 3.4.3. Reflectivity

Reflectivity is the compound’s property that tells us that when light strikes it, it reflects the light back to us. How much of this radiation will hit a compound’s surfaces? It will reverse to its former position if it reaches a certain quantity. In optical physics, the term “*R*(*w*)” is capitalized to indicate reflectivity. Utilizing the dielectric function’s imaginary component, the reflection coefficient was calculated:

(12) $$\delta (\omega )=\frac{2w_{ev}\hbar \omega }{E_{o}}$$

**Figure 8:**
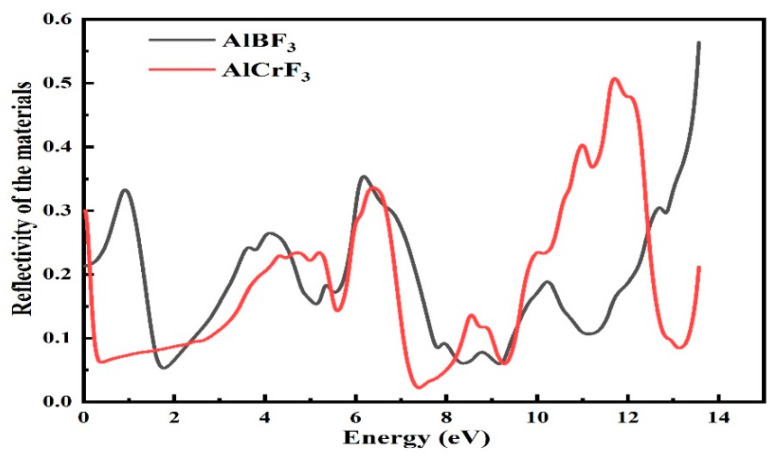
Calculated reflectivity coefficient *R*(*ω*) for compounds.

Figure 8 shows that the value of static reflection *R*(0) for the compound AlCrF_3_ was almost similar to *R*(0) = 0.3 at zero photon energy, whereas for AlBF_3_ it was 0.23. The compound AlBF_3_ had the highest radiation reflection, with *R*(*w*) = 0.34, 0.35, and 0.55 at 0.5, 6.3, and 13.5 eV. For the compound AlCrF_3_, the highest peak reflectivity values were 0.30 at 0.0 eV, 0.34 at 6.3 eV, and 5.5 at 13.5 eV. Figure 8 shows that these materials had lower reflection values.

The coefficient is measured in terms of energy. Because these compounds are used by professionals as anti-reflecting coaters, their low reflection coefficient score—especially in the visible and infrared sections—suggests that they appear transparent in these regions. Due to their employment by scientists as anti-reflecting coaters, the small score of this phrase, mostly in the visible and infrared quotas, shows that these substances exhibit transparency in these areas.

## 4. Conclusions

The current study used TB-MBJ potential approximations to examine the structural, optical, elastic, and electrical features of AlMF_3_ (M = Cr, B) fluoroperovskites. In general, the PBE scheme underestimated the value of the band gap, and it was shown that a U value of 4 eV for Cr atoms produces much better results. The equilibrium lattice constants of the AlCrF_3_ were found to be between 7.96 and 7.80, whereas those of the AlBF_3_ were found to be between 4.6999 and 4.70. The elastic properties of the ECs, anisotropy factor, bulk modulus, Poisson’s ratio, Young’s modulus, and Pugh’s ratio were all as expected. The Pugh (*B/G*) ratio indicated ductility in the substances under examination; the estimated Poisson ratio values supported the ductility of the material. Both compounds were brittle, stiff, and anisotropic, and they had ionic bonding. AlCrF_3_ showed indirect band gap behavior at the (X-M) symmetry point according to our observations, but AlBF_3_ had overlapping of the states, according to our calculations. Using the GGA + U method for AlCrF_3_ resulted in it having a half-metallic nature. When the calculated results were compared to existing experimental and theoretical data, they were found to be consistent.

## Figures and Tables

**Table 1:** Optimized structural characteristics of TlMF_3_ (M = Au, Ga) derived using Birch-energy Murnaghan’s vs. volume.

Compounds	ao (Lattice Constant in Å)	B (Bulk Modulus in GPa)	B’ (Derivative of Bulk Modulus)	V0 (Ground State Volume in a.u3)	E0 (Ground State Energy in Ry)
**AlCrF_3_**	7.96	89.57	4.80	457.18	−3186.77
**AlBF_3_**	7.70	81.15	4.66	401.27	−1134.47

**Table 2:** Simulated mechanical parameters of AlMF_3_ (M = Cr, B) using the IRelast package.

Compounds	AlCrF_3_	AlBF_3_
***C_11_***	*79.11*	*77.00*
***C_12_***	*44.85*	*94.22*
***C_44_***	*11.86*	*31.92*
***B***	*89.57*	*81.15*
***G***	*13.74*	*10.22*
***E***	*40.80*	*45.74*
***A***	*0.69*	*3.71*
***v***	*0.46*	*0.46*
***B/G***	*12.51*	*16.83*

## Data Availability

All data are available within this publication.
